# Pathological diagnosis of thyroid nodules based on core needle biopsies: comparative study between core needle biopsies and resected specimens in 578 cases

**DOI:** 10.1186/s13000-019-0786-4

**Published:** 2019-02-02

**Authors:** Yan Xiong, Limin Yan, Lin Nong, Yalin Zheng, Ting Li

**Affiliations:** 1 Department of Pathology, Peking University First Hospital, 7 Xishiku Street, Xicheng District, Beijing, 100034 China; 2 Department of Pathology, Tangshan Gongren Hospital, 27 Wenhua Road, Lubei District, Hebei, 063000 China

**Keywords:** Thyroid nodule, Core needle biopsy, Pathological diagnosis

## Abstract

**Background:**

Pathological diagnosis based on core needle biopsy (CNB) should be different from a resected specimen because it is difficult to apply the histological criteria established for resected specimens to CNB due to sampling limitations. A pathological classification for thyroid nodule on CNB was first proposed by the Korean Group in 2015. The objective of this study was to test the reliability and clinical value of this proposal.

**Methods:**

According to the Korean proposal, the CNB diagnoses were categorized into unsatisfactory, benign, indeterminate, follicular neoplasm, suspicious for malignancy and malignant. A comparative study between the diagnoses of CNB and resected specimens was performed.

**Results:**

The consistency was moderate (κ = 0.448). Combined indeterminate, suspicious for malignancy and malignant into a single group collectively referred to as “malignant” with the remaining merged into “others”, CNB demonstrated a 95.93% sensitivity, 97.30% specificity, 62.07% accuracy, 99.81% positive predictive value (PPV) and 62.07% negative predictive value (NPV) for preoperative malignancy evaluation.

**Conclusions:**

The Korean proposal for pathological classification of thyroid nodules on CNB is objective, operable and highly valuable.

## Background

Thyroid nodule is a common disease of the endocrine system. The prevalence is 20 to 76% in the Chinese population as identified by high-resolution ultrasound, and 5 to 15% of nodules are malignant [1]. It is very important to screen these malignant cases for further treatment.

At present, the biopsy techniques for thyroid nodules used in practice include fine needle biopsy (FNB) and core needle biopsy (CNB). FNB aspirates cells for cytological examination with 22-27G needles, while CNB punctures tissues for histological examination with 18-21G needles.

FNA was applied to the preoperative diagnosis of thyroid nodules for the first time in 1960s and has been the standard diagnostic tool since the 1980s [2, 3]. CNB was first used in preoperative diagnosis of thyroid nodules in the 1990s. CNB has not been widely used for many years because of the pain, tolerability and complications associated with the operation [4]. However, with advances in CNB devices (i.e., spring-activated core needles) and the development of high-resolution ultrasound, these disadvantages have been significantly reduced. Recently, several large single-center studies have shown no significant differences between FNB and CNB in terms of pain, tolerability, or complications [5, 6]. In this case, the advantage of CNB for obtaining a large amount of tissue and providing more information on histological structures is highlighted. The National Cancer Institute, American Association of Clinical Endocrinologists/American College of Endocrinology/Associazione Medici Endocrinologi (AACE/ACE/AME), and the Korean Society of Thyroid Radiology (KSThR) have proposed CNB for thyroid nodules with previous nondiagnostic FNA results [7–9]. Some publications even indicate that CNB may be a feasible and effective diagnostic tool for initially detected thyroid nodules [10–12].

The application of CNB for preoperative diagnosis of thyroid nodules brings new challenges for pathologists. The lack of widely accepted pathological classification has led to inefficient communication between pathologists and clinicians and even misinterpretation of pathological results. Therefore, an effective pathological classification for thyroid nodules on CNB is urgently required by pathologists and clinicians. Of the very limited publications focusing on this issue, the majority are from Korea, the most important of which was the proposal of the Korean Endocrine Pathology Thyroid Core Needle Biopsy Study Group in 2015 [13]. The terminology and criteria for thyroid nodules on CNB specimen were defined for the first time in the proposal. This is a significant advancement, but the objectivity, accuracy and clinical value of this proposal should be tested in more centers and more case studies around the world to further improve and optimize the criteria based on worldwide data.

We retrieved 578 cases of thyroid nodules from Peking University First Hospital and classified them according to the Korean proposal. We tested the reliability of the Korean proposal and evaluated its clinical value by comparing classifications of a CNB sample to a resected specimen from the same nodule.

## Methods

### Patients and samples

Patients with thyroid nodules treated at Peking University First Hospital from January 2015 to December 2017 were reviewed. Cases of thyroid follicular lesions with both CNB and resected specimens were retrieved. CNB was used as the first-line preoperative diagnosis in all cases without prior FNB and was performed according to the recommendations from publications [10, 14].

### Pathological review

All slides were separately reviewed by two pathologists blinded to the original diagnosis. Cases with inconsistent diagnoses were reviewed and agreement was achieved by discussion.

### Six-classification method for CNB specimens

**Fig. 1:**
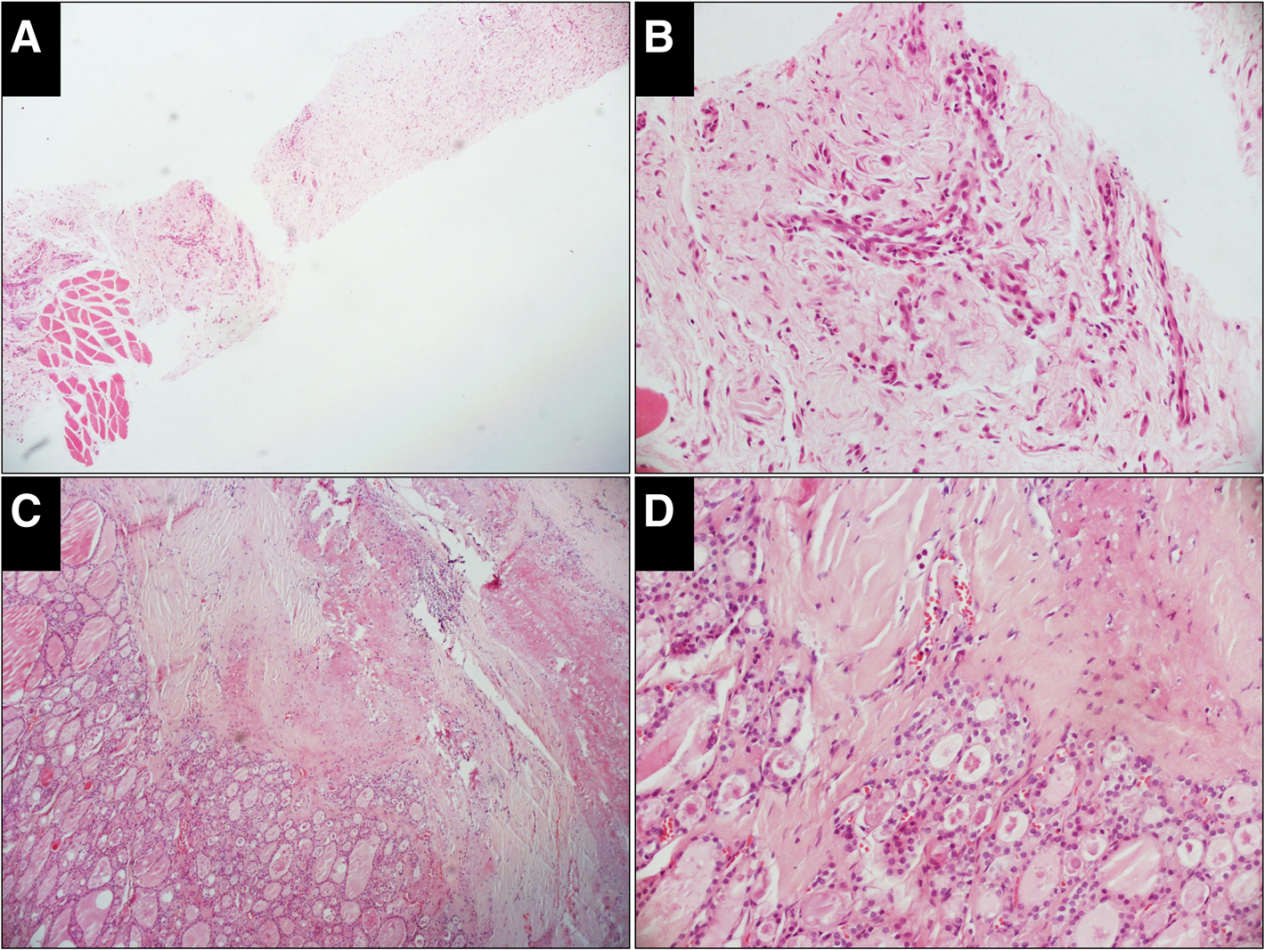
Case classified as unsatisfactory in the CNB specimen while benign in the resected specimen (H&E). **a,** The CNB specimen consists of an acellular fibrotic lesion with a few striated muscles at one end (× 50). **b,** High-power view of the fibrotic lesion shows no thyroid follicles (× 200). **c** (× 50), **d** (× 200), Thyroid follicles in the resected specimen are almost normal with fibrotic tissue around it

**Fig. 2:**
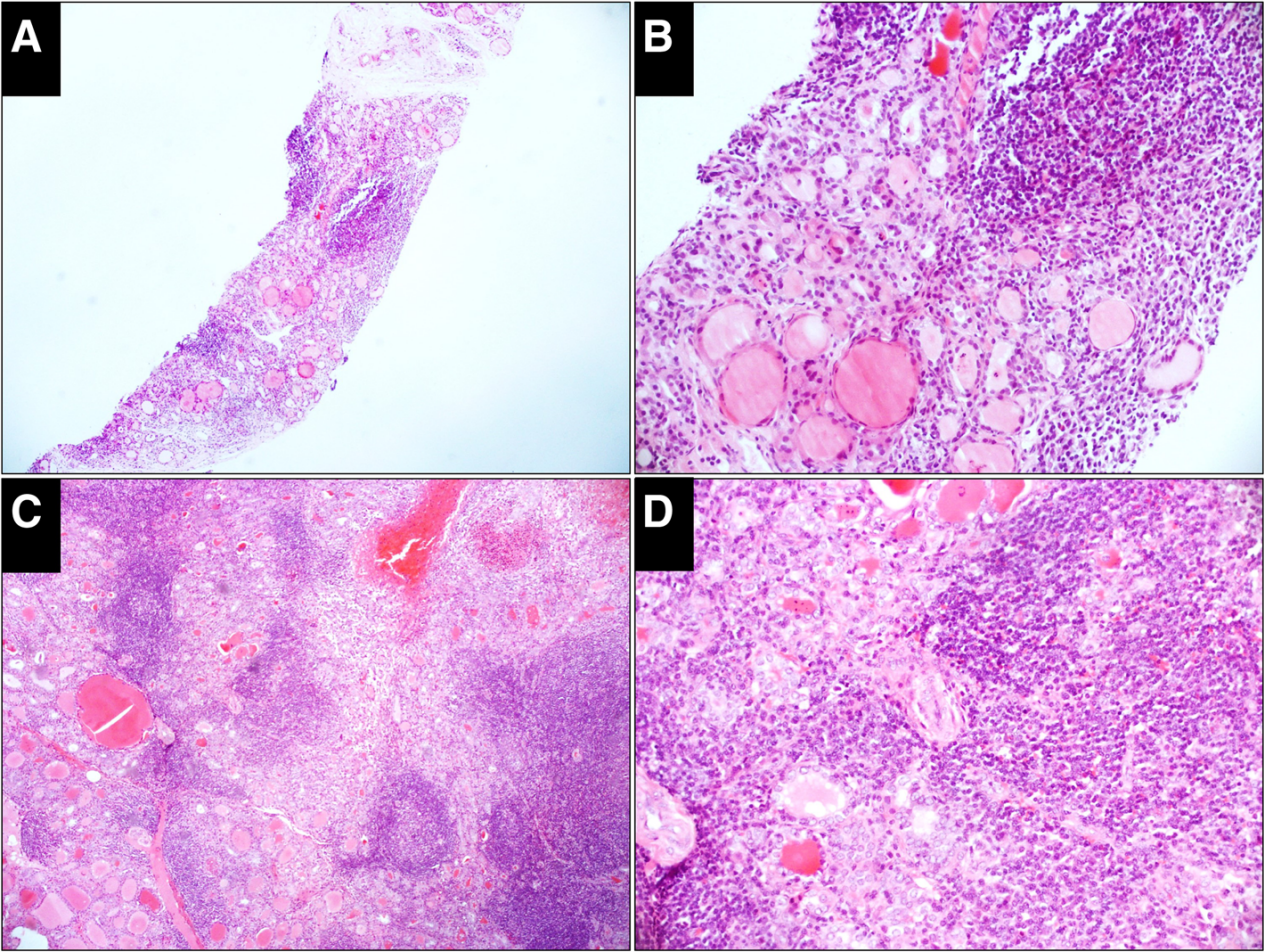
Case classified as benign both in the CNB and resected specimens (H&E). a, The CNB specimen consists of thyroid follicles and many lymphoid tissues with lymphatic follicles (× 50). **b**, Thyroid follicles are atrophic with oncocytic metaplasia (× 200). **c** (× 50), **d** (× 200), The change in the thyroid follicles and the proliferation of lymphoid tissues in the resected specimen is the same as that in the CNB specimen

**Fig. 3:**
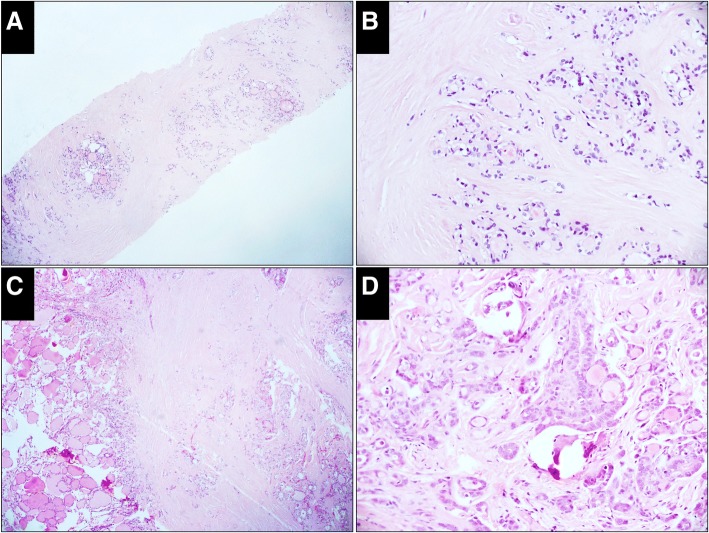
Case classified as indeterminate in the CNB specimen but malignant in the resected specimen (H&E). **a**, The CNB specimen consists of hyalinized fibrous tissue with embedded microfollicles (× 50). **b**, High-power view of the microfollicle cells shows enlarged nuclei but neither glassy nor irregular membrane (× 200). **c**, A circumscribed sclerosing nodule is prominent in the resected specimen, in which microfollicles, small tubules and elongated glands are embedded (× 50). **d,** High-power view of the cells shows nuclei with features of papillary thyroid carcinoma (× 200)

**Fig. 4:**
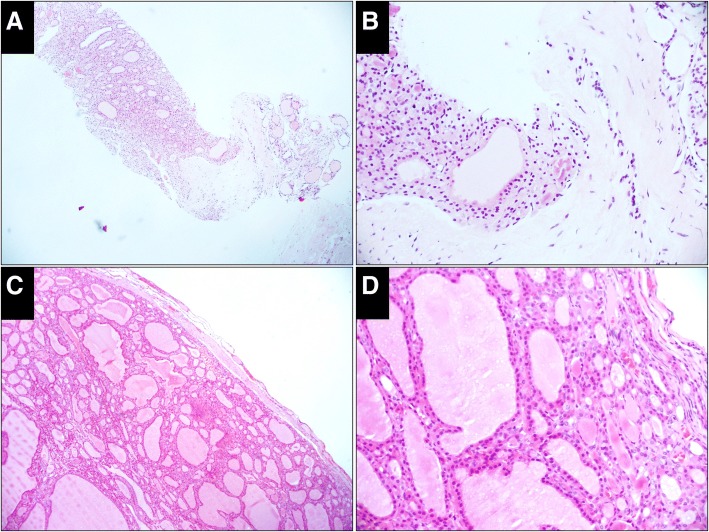
Case classified as follicular neoplasm in both the CNB and resected specimens (H&E). **a**, The CNB specimen shows a microfollicular proliferative lesion with a fibrous capsule separating it from the normal follicles (× 50). **b**, High-power view shows proliferative follicles on the left of the capsule and the normal follicles on the right (× 200). **c**, An encapsulated follicular nodule with a complete thin capsule is prominent in the resected specimen. No capsular and vascular infiltration is detected (× 50). **d,** High-power view of the cells shows nuclei without features of papillary thyroid carcinoma (× 200)

**Fig. 5:**
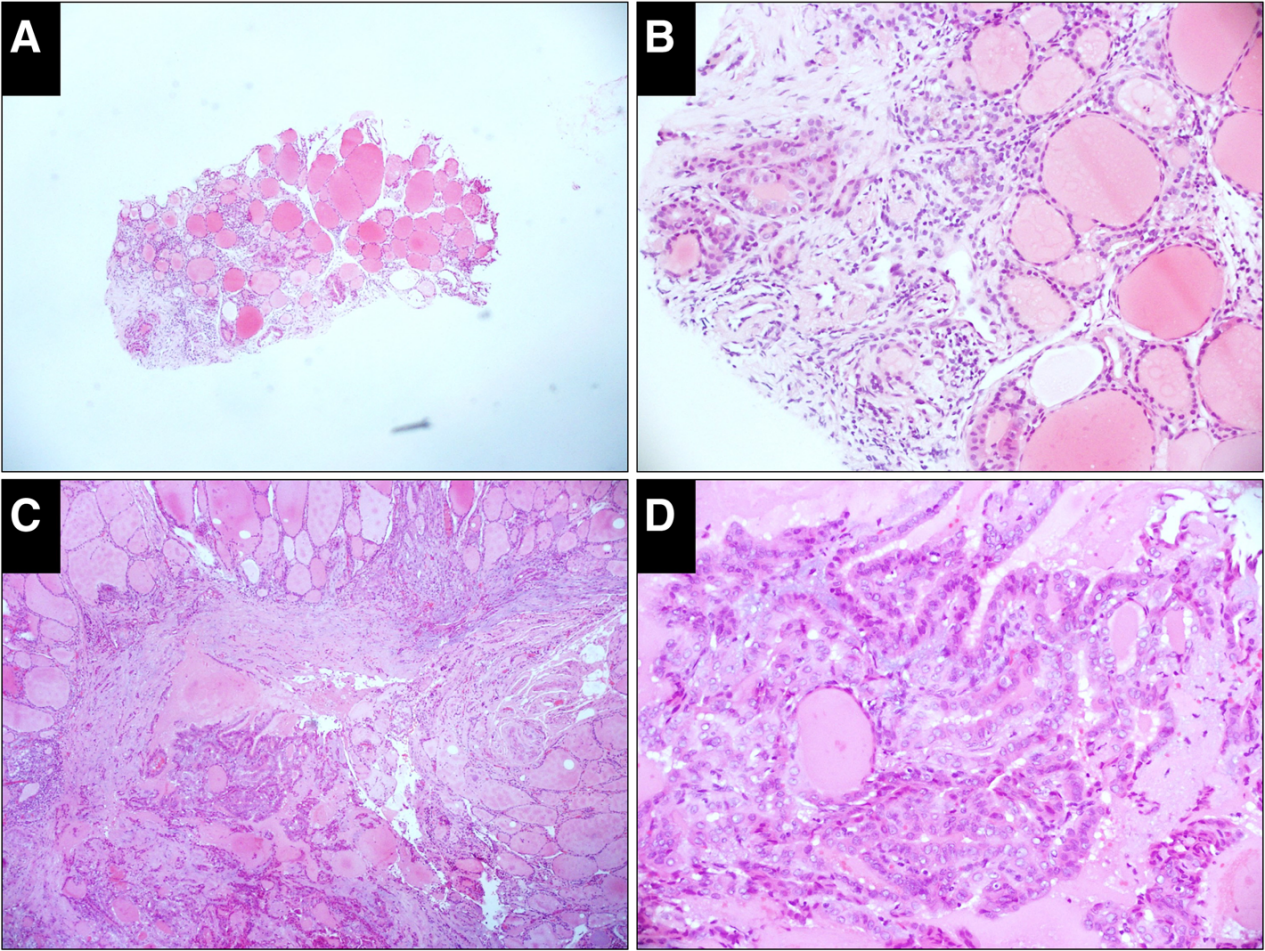
Case classified as suspicious for malignancy in the CNB specimen but malignant in the resected specimen (H&E). **a**, The CNB specimen is mainly composed of normal thyroid follicles except for a focal fibrotic lesion at the end (× 50). **b**, High-power view of the fibrotic lesion shows several embedded small tubules and the cell nuclei possess the features of papillary thyroid carcinoma (× 200). **c**, A circumscribed sclerosing nodule is prominent in the resected specimen, with a number of small tubules and elongated glands (× 50). **d**, High power view of the cells shows nuclei with the features of papillary thyroid carcinoma (× 200)

**Fig. 6:**
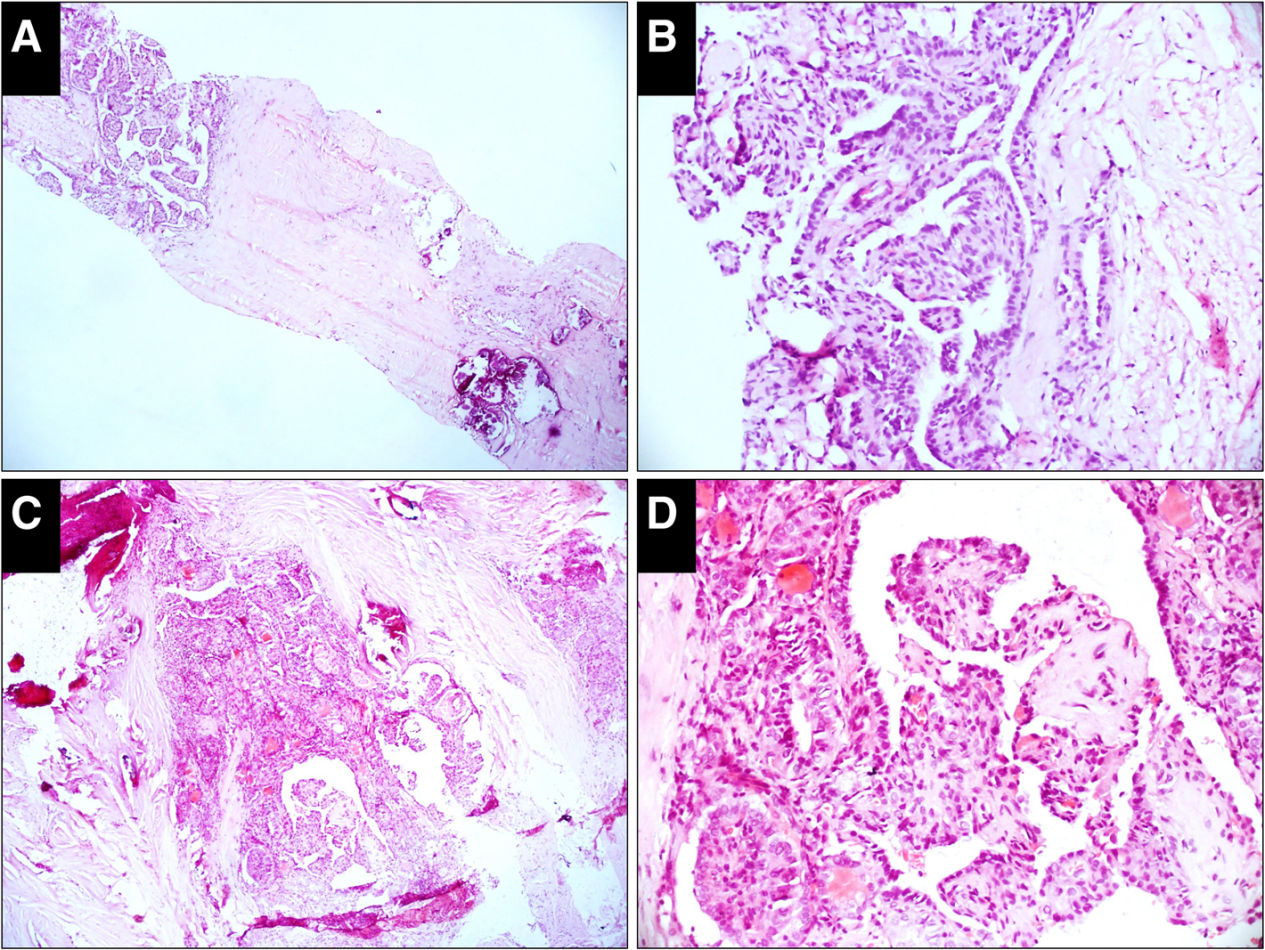
Case classified as malignant in both the CNB and resected specimens (H&E). **a**, Papillary pattern with calcification is embedded in the hyalinized collagen (× 50). **b**, High-power view of the papillae shows the cells with typical nuclear features of papillary thyroid carcinoma (× 200). **c**, An infiltrating tumor with stromal fibrosis is prominent in the resected specimen. The major architecture of the tumor is a papillary and elongated glandular pattern (× 50). **d,** High-power view of the papillae shows the cells with the nuclear features of papillary thyroid carcinoma (× 200)

According to the Korean proposal (Table 1) [13], diagnoses of CNB samples were divided into the following six categories: I. Unsatisfactory (Fig. 1); II. Benign (Fig. 2); III. Indeterminate (Fig. 3), including indeterminate follicular lesion with nuclear atypia and/or architectural atypia; IV, Follicular neoplasm (Fig. 4); V, Suspicious for malignancy (Fig. 5); and VI, Malignant (Fig. 6).

**Table 1:** Diagnostic Categories of Thyroid Core Needle Biopsy Proposed by The Korean Endocrine Pathology Thyroid Core Needle Biopsy Study Group

I. Nondiagnostic or unsatisfactory	
• Normal thyroid tissue only	
• Extrathyroid tissue only (e.g., skeletal muscle, mature adipose tissue)	
• A virtually acellular specimen	
• Acellular/paucicellular fibrotic nodule	
• Blood clot only	
• Other	
II. Benign lesion	
• Benign follicular nodule or consistent with a benign follicular nodule	
• Hashimoto’s thyroiditis	
• Granulomatous (subacute) thyroiditis	
• Nonthyroidal lesion (e.g., parathyroid lesions, benign neurogenic tumors, benign lymph node)	
• Other	
III. Indeterminate lesion	
IIIA. Indeterminate follicular lesion with nuclear atypia	
• Follicular proliferative lesions with focal nuclear atypia	
• Follicular proliferative lesions with equivocal or questionable nuclear atypia	
• Atypical follicular cells embedded in a fibrotic stroma	
IIIB. Indeterminate follicular lesion with architectural atypia	
• Microfollicular proliferative lesion lacking a fibrous capsule or the adjacent nonlesional tissue in the specimen	
• Solid or trabecular follicular lesion lacking a fibrous capsule or the adjacent nonlesional tissue in the specimen	
• Macrofollicular proliferative lesion with a fibrous capsule	
• Hürthle cell proliferative lesion lacking a fibrous capsule or the adjacent nonlesional tissue in the specimen	
IIIC. Other indeterminate lesions	
IV. Follicular neoplasm or suspicious for a follicular neoplasm	
• Microfollicular proliferative lesion with a fibrous capsule	
• Mixed microfollicular and normofollicular proliferative lesion with a fibrous capsule	
• Solid/trabecular follicular proliferative lesion with a fibrous capsule	
• Hürthle cell proliferative lesion with a fibrous capsule	
• Follicular neoplasm with focal nuclear atypia	
V. Suspicious for malignancy	
• Suspicious for papillary carcinoma, medullary carcinoma, poorly differentiated carcinoma, metastatic carcinoma, lymphoma, etc.	
VI. Malignant	
• Papillary thyroid carcinoma, poorly differentiated carcinoma, undifferentiated (anaplastic carcinoma), medullary thyroid carcinoma, lymphoma, metastatic carcinoma, etc.

### Six-classification method for resected specimens

Referring to the six-classification method for CNB, diagnoses of resected specimens were also grouped into six categories as follows: I. Unsatisfactory, i.e., no thyroid tissue in the specimen; II. Benign lesion, including nodular hyperplasia, thyroiditis and benign neoplasm except follicular adenoma; III. Indeterminate lesion; IV. Follicular neoplasm, including follicular adenoma and follicular carcinoma; V. Suspicious for malignancy, i.e., suspicious malignant neoplasms of follicular epithelium except follicular carcinoma; and VI. Malignant, i.e., malignant neoplasms of follicular epithelium except follicular carcinoma.

### Two-classification method for CNB and resected specimens

To simplify the categories for statistical analysis, the classifications were reduced from six to two as follows: two-classification method A, which combined V and VI into a single group collectively referred to as “malignant” with the remaining four categories merged into “others”; and two-classification method B, which combined III, V and VI into a single group collectively referred to as “malignant” with the remaining three categories merged into “others”.

### Statistical analysis

Fleiss’s kappa was used to test the consistency between the CNB and resected specimen classifications. A κ value < 0.00 indicates poor consistency, 0.01–0.20 indicates slight consistency, 0.21–0.40 indicates fair consistency, 0.41–0.60 indicates moderate consistency, 0.61–0.80 indicates substantial consistency, and 0.81–1.00 indicates almost perfect consistency [15].

Based on the data obtained by the two-classification method and taking the resected specimen classifications as the gold standard, the sensitivity, specificity, accuracy, positive predictive value (PPV) and negative predictive value (NPV) of CNB for preoperative malignancy evaluation were calculated. The formulas were as follows:

Sensitivity = Number of cases classified as “malignant” based on both the CNB and resected specimens/ Number of cases classified as “malignant” based on both the CNB and the resected specimens + Number of cases classified as “others” based on the CNB specimen but as “malignant” based on the resected specimen × 100%.

Specificity = Number of cases classified as “others” based on both the CNB and the resected specimens/ Number of cases classified as “others” based on both the CNB and the resected specimens + Number of cases classified as “malignant” based on the CNB specimen but as “others” based on the resected specimens × 100%.

Accuracy = Number of cases classified as “malignant” based on both the CNB and the resected specimens + Number of cases classified as “others” based on both the CNB and the resected specimens / Number of all cases × 100%.

PPV = Number of cases classified as “malignant” based on both the CNB and the resected specimens/ Number of cases classified as “malignant” based on both the CNB and the resected specimens + Number of cases classified as “malignant” based on the CNB specimen but as “others” based on the resected specimens × 100%.

NPV = Number of cases classified as “others” based on both the CNB and the resected specimens/ Number of cases classified as “others” based on both the CNB and the resected specimens + Number of cases classified as “others” based on the CNB specimen but as “malignant” based on the resected specimens × 100%.

## Results

### Patients

The study included 578 patients. Of them 146 were males and 432 were females, ranging in age from 20 to 81 years old with a median age of 38 years old. All of patients had no obvious discomfort during operation and no hoarseness after operation, only one patient presented with perithyroidal hemorrhage and achieved remission without any treatment.

### Results from the six-classification method

CNB specimen results: Of the 578 cases, 1 (0.17%) was unsatisfactory, 40 (6.92%) were benign, 31 (5.36%) were indeterminate, 17 (2.94%) were follicular neoplasm, 26 (4.50%) were suspicious for malignancy, and 463 (80.10%) were malignant (Table 2). Resected specimen results: Of the 578 cases, 21 (3.63%) were benign, 15 (2.60%) were follicular neoplasm, 3 (0.52%) were suspicious for malignancy, 539 (93.25%) were malignant, and none was classified as unsatisfactory or indeterminate (Table 2).

**Table 2:** Comparison Between the Classification of Thyroid Nodules based on CNB and Resected Specimens from Six-Classification Method

CNB Specimen, No.	Resected Specimen, No.							κ
	I	II	III	IV	V	VI	Total	
I	0	1	0	0	0	0	1	0.448
II	0	19	0	0	1	20	40	
III	0	0	0	1	2	28	31	
IV	0	0	0	16	0	1	17	
V	0	0	0	0	0	26	26	
VI	0	0	0	0	0	463	463	
Total	0	20	0	17	3	538	578	

CNB, core needle biopsy

### Results from the two-classification method

CNB results from method A: Of the 578 cases, 490 (84.78%) were classified as “malignant” and 88 (15.22%) as “others” (Table 3). CNB results from method B: Of the 578 cases, 519 (89.79%) were classified as “malignant” and 59 (10.21%) as “others” (Table 3). The resected specimen results from method A and B were the same. Of the 578 cases, 541 (93.60%)were classified as “malignant” and 37 (6.40%) as “others” (Table 3).

**Table 3:** Comparison Between the Classification of Thyroid Nodules based on CNB and Resected Specimens from Two-Classification Method

		Resected specimen, No.					
CNB specimen, No.		A		B		Total	κ
		Malignancy	Others	Malignancy	Others		
A	Malignancy	489	0			489	0.546
	Others	52	37			89	
B	Malignancy			519	1	520	0.737
	Others			22	36	58	
Total		541	37	541	37		

*CNB* core needle biopsy

### Comparison between the CNB and resected specimens results from the six-classification method

The CNB results were consistent with the resected specimen results in 498 cases and inconsistent in 80 cases. Of the 80 inconsistent cases, 1 was classified as unsatisfactory based on the CNB specimen but benign based on the resected specimen; 1 was classified as benign based on the CNB specimen but suspicious for malignancy based on the resected specimen; 20 were classified as benign based on the CNB specimens but malignant based on the resected specimens; 1 was classified as indeterminate based on the CNB specimen but as follicular neoplasm based on the resected specimen; 2 were classified as indeterminate based on the CNB specimens but suspicious for malignancy based on the resected specimens; 28 were classified as indeterminate based on the CNB specimens but malignant based on the resected specimens; 1 was classified as follicular neoplasm based on the CNB specimen but malignant based on the resected specimen; and 26 were classified as suspicious for malignancy based on the CNB specimens but malignant based on the resected specimens (Table 2). The consistency between the CNB and resected specimens classification is moderate (κ = 0.448).

### Comparison between the CNB and resected specimens results from the two-classification method a

The CNB specimen classification was consistent with the resected specimen classification in 526 cases and inconsistent in 52 cases. All 52 inconsistent cases were classified as “others” based on the CNB specimens but as “malignant” based on the resected specimens (Table 3). The consistency between the classification of the CNB and resected specimens is moderate (κ = 0.546). Taking the classification of resected specimens as the gold standard, CNB demonstrates a 90.39% sensitivity, 100.00% specificity, 91.00% accuracy, 100.00% PPV, and 41.57% NPV for preoperative evaluation of thyroid nodules (Table 4).

**Table 4:** Value of CNB for Preoperatively Evaluating Malignancy of Thyroid Nodules

	Sensitivity, %	Specificity, %	Accuracy%	PPV, %	NPV, %
Two-classification method A	90.39	100.00	91.00	100.00	41.57
Two-classification method B	95.93	97.30	96.02	99.81	62.07

PPV, positive predictive value; NPV, negative predictive value

### Comparison between the CNB and resected specimen results from the two-classification method B

The CNB specimen classification was consistent with the resected specimen classification in 555 cases and inconsistent in 23 cases. Of the 23 inconsistent cases, 1 was classified as “malignant” based on the CNB specimen but was classified as “others” based on the resected specimen, and 22 were classified as “others” based on the CNB specimens but were classified as “malignant” based on the resected specimens (Table 3). The consistency between the classification of the CNB and resected specimens is substantial (κ = 0.737). Taking the classification of resected specimens as the gold standard, CNB demonstrates a 95.93% sensitivity, 97.30% specificity, 62.07% accuracy, 99.81% PPV, and 62.07% NPV for preoperative evaluation of thyroid nodules (Table 4).

## Discussion

Pathological diagnosis of tumors with CNB specimens is unique. The reason is that, on the one hand, it is very different from the cytology observed with FNB and, therefore, either the diagnostic criteria or terminology should be different, and on the other hand, due to sampling limitations, it is difficult to apply the histological criteria established for resected specimens to CNB. In practice, the lack of uniform criteria and terminology often leads to deficiencies in the rigor and standardization of reports, consequently reducing the clinical value of CNB. Therefore, pathologists and clinicians urgently need a pathological classification that is not only based on the histological characteristics of CNB specimens but also objective, operable and highly valuable. This claim was first realized in the pathological diagnosis of lung cancer. The 2015 edition of WHO classification of tumours of the lung established the terminology and criteria for non-small cell carcinoma on CNB independently of resected specimens for the first time [16]. This system has been widely welcomed by pathologists and clinicians in the last 3 years. The successful experience has prompted the international pathology community to understand the specificity of the diagnosis for CNB specimens. After lung cancer, various studies have been performed in other subspecialties.

Unlike the preoperative diagnosis of lung nodules, the preoperative diagnosis of thyroid nodules around the world is still mainly dependent on FNB, although many publications have confirmed the safety of CNB for thyroid nodules [6]. In China, CNB is applied in the diagnosis of thyroid nodules in only a few medical centers. Therefore, research on the pathological diagnosis of thyroid nodules by CNB is relatively insufficient. There are very limited publications and the majority are from Korea, the most important of which is the proposal of the Korean Endocrine Pathology Thyroid Core Needle Biopsy Study Group 2015 [13]. The terminology and criteria for CNB thyroid nodule specimen were standardized for the first time in this proposal. This is a significant study, and the objectivity, accuracy and clinical value of the findings should be tested in more centers and more case studies around the world to improve and optimize the criteria based on a larger amount of data.

In our study, of 578 CNB samples, only 1 (0.17%) was classified as unsatisfactory, and 31 (5.36%) were classified as indeterminate. Compared to the unsatisfactory rate (5–17%) and indeterminate rate (10–30%) of FNB published in papers [17–19], the rate of CNB is much lower. There were 28 cases classified as indeterminate based on CNB that were classified as malignant in resected specimens. In all of these cases, the histological pattern of the CNB specimens was entirely follicle, while that of resected specimens was a mixture of follicle, tubule and papillae. This demonstrates that sampling limitation is the main reason why the cases classified as malignant on resected specimens were classified as indeterminate based on CNB. Nevertheless, the atypia of the histological pattern and cell morphology of the CNB specimens highly suggested that the cases were malignant. There were 26 cases classified as suspicious for malignancy based on CNB that were classified as malignant in the resected specimens. Of these cases, a very few fragments of papillae were found in CNB specimens in 10 cases, and several microfollicles composed of cells with nuclear features of papillary thyroid carcinoma were present in 16 CNB specimens. Although, the components with the histological characteristics of papillary thyroid carcinoma did exist in CNB in these cases, they were classified as suspicious for malignancy only because the quantity was insufficient to make a definitive diagnosis. Therefore, sampling limitation is also the main reason that cases classified as malignant on resected specimens were classified as suspicious for malignancy on CNB.

By two-classification method A, the consistency between CNB classification and resected specimen classification is moderate (κ = 0.546) and CNB shows 90.39% sensitivity, 100.00% specificity, 91.00% accuracy, 100.00% PPV and 41.57% NPV for the preoperative evaluation of the malignancy of thyroid nodules. Therefore, we recommend that cases classified as suspicious for malignancy and malignant based on CNB be treated as malignant. By two-classification method B, the consistency between classification on CNB and resected specimens is substantial (κ = 0.737), and CNB shows 95.93% sensitivity, 97.30% specificity, 62.07% accuracy, 99.81% PPV and 62.07% NPV for the preoperative evaluation of the malignancy of thyroid nodules. Compared to method A, although 0.19% of the patients were at risk of over treatment, method B reduces the rate of missed diagnosis of malignancy by 20.50% and increases the accuracy of overall evaluation of malignancy by 5.02%. Thus, we think it may be the most beneficial strategy for cases classified as indeterminate, suspicious for malignancy and malignant based on CNB to be treated as malignant. Of the 40 cases classified as benign on CNB, 19 were classified as benign, 20 were classified as malignant and 1 was classified as suspicious for malignancy on resected specimens. A study of the morphology of the inconsistent cases shows that although the malignant components are obvious in resected specimens, they are absent in CNB. Failure to obtain tissue from the lesion is the only reason for the discrepancy. Therefore, cases classified as benign by CNB but considered malignant or suspicious for malignancy by ultrasound should be followed up closely and require rebiopsy.

FNB is incapable of differentiating follicular neoplasm from nodular hyperplasia, while CNB is very useful in making this distinction. Of 17 cases classified as follicular neoplasm with the resected specimens, 16 were classified as follicular neoplasm and 1 was classified as indeterminate by CNB. Therefore, cases classified as follicular neoplasm by CNB should be treated with surgical operation. The capsular and vascular infiltration should be detected microscopically to further clarify whether the lesion is benign or malignant.

## Conclusions

CNB is a safe and effective method for the preoperative diagnosis of thyroid nodules. The proposal of the Korean Endocrine Pathology Thyroid Core Needle Biopsy Study Group suggestion 2015 is objective, operable and highly valuable for pathological classification of thyroid nodules on CNB. Under this framework, the inaccuracy and limitations of biopsy is the main cause of inconsistency between diagnosis based on CNB and resected specimens. The most beneficial strategy for cases classified as indeterminate, suspicious for malignancy and malignant on CNB may be to treat them as malignant. Cases classified as benign by CNB but considered malignant or suspicious for malignancy by ultrasound should be followed up closely and require rebiopsy. Cases classified as follicular neoplasm by CNB should be treated with surgical operation, and the capsular and vascular infiltration should be detected microscopically to further clarify whether the lesion is benign or malignant.

## Abbreviations

AACE: American Association of Clinical Endocrinologists
ACE: American College of Endocrinology
AME: Associazione Medici Endocrinologi
CNB: Core needle biopsy
FNB: Fine needle biopsy
KSThR: Korean Society of Thyroid Radiology
NPV: Negative predictive value
PPV: Positive predictive value
